# How do patients with chronic illnesses respond to a public health crisis? Evidence from diabetic patients in Japan during the COVID-19 pandemic

**DOI:** 10.1016/j.ssmph.2021.100961

**Published:** 2021-11-15

**Authors:** Masataka Harada, Takumi Nishi, Toshiki Maeda, Kozo Tanno, Naoyuki Nishiya, Hisatomi Arima

**Affiliations:** a Department of Industrial Economics, Faculty of Economics, Fukuoka University, Fukuoka, Japan; b Department of Research Planning and Information Management, Fukuoka Institute of Health and Environmental Sciences, Fukuoka, Japan; c Department of Preventive Medicine and Public Health, Faculty of Medicine, Fukuoka University, Fukuoka, Japan; d Department of Hygiene and Preventive Medicine, Iwate Medical University, Iwate, Japan; e Iwate Tohoku Medical Megabank Organization, Iwate Medical University, Iwate, Japan; f Division of Integrated Information for Pharmaceutical Sciences, Department of Clinical Pharmacy, Iwate Medical University School of Pharmacy, Iwate, Japan

**Keywords:** COVID-19, Diabetes, Doctor visits, Japan, Socioeconomic disparities

## Abstract

How do people change their healthcare behavior when a public health crisis occurs? Within a year of its emergence, coronavirus disease 2019 (COVID-19) has gradually infiltrated our lives and altered our lifestyles, including our healthcare behaviors. In Japan, which faces China across the East China Sea and accepted 924,800 Chinese tourists in January 2020, the emergence and spread of COVID-19 provides a unique opportunity to study people's reactions and adaptations to a pandemic.

Patients with chronic illnesses who require regular doctor visits are particularly affected by such crises. We focused on diabetic patients whose delay in routine healthcare invites life-threatening complications and examined how their patterns of doctor visits changed and how demographic, socioeconomic, and vital factors disparately affected this process. We relied on the insurance claims data of a health insurance association in Tokyo. By using panel data of diabetic patients from April 2018 to September 2020, we performed visual investigations and conditional logistic regressions controlling for all time-invariant individual characteristics.

Contrary to the general notion that the change in healthcare behavior correlates with the actual spread of the pandemic, the graphical and statistical results both showed that diabetic patients started reducing their doctor visits during the early stage of the pandemic. Furthermore, a substantial decrease in doctor visits was observed in women, and large to moderate reductions were seen in patients who take insulin and are of advanced age, who are at high risk of developing severe COVID-19. By contrast, no differentiated effect was found in terms of income status. We further investigated why a change in pattern occurred for each subgroup.

The patterns of routine healthcare revealed by this study can contribute to the improvement of communication with the target population, the delivery of necessary healthcare resources, and the provision of appropriate responses to future pandemics. (299 words).

## Highlights

• Diabetic patients started decreasing doctor visits since the emergence of COVID-19.
• Female patients reduced doctor visits most rapidly regardless of their labor status.
• Patients at high risk of severe COVID-19 set very long interval in doctor visits.

## 1 Introduction

Since coronavirus disease 2019 (COVID-19) was first reported in Wuhan, the infection has damaged and ruined people's lives worldwide. Despite the efforts of many countries to contain this contagious disease, no promising treatment has been found, and COVID-19 is still spreading throughout the world. Most young, healthy people infected with the new coronavirus experience only a mild to moderate respiratory illness and will recover without any special treatment (WHO n.d.). However, the elderly and people with underlying medical problems, such as cardiovascular disease, diabetes, chronic respiratory disease, and malignancy, are more likely to develop severe pneumonia (WHO n.d.; Onder et al., 2020).

Although chronic diseases require regular visits, monitoring, and care to control, there is growing concern that COVID-19 might invite care avoidance or “temporary disruptions in routine and non-emergency medical care access” (Czeisler et al., 2020, Moroni et al., 2020). A study reported that 31.5% of US adults avoided routine medical care during the pandemic because of COVID-19 (Czeisler et al., 2020). In addition, people with underlying medical conditions are more likely to avoid routine care visits (Czeisler et al., 2020). Therefore, medical care avoidance triggered by COVID-19 can exacerbate chronic diseases and other related symptoms.

At the same time, how severely a dropout from routine medical care affects one's state of the disease varies by the type of chronic illness. Therefore, this study focused on the healthcare behaviors of diabetic patients. Diabetes is one of the most common life-threatening chronic diseases and one of the primary causes of serious complications such as atherosclerotic cardiovascular disease and microvascular disease (i.e., retinopathy, nephropathy, and neuropathy), particularly when patients miss adequate care management (American Diabetes Association 2021a, 2021b). Diabetic patients are also susceptible and vulnerable to COVID-19 (Fang et al., 2020) and may withhold or reduce their visits for routine healthcare. However, studies examining the healthcare behavior of diabetic patients are still scarce. Therefore, it is worth exploring their changing patterns of routine medical care during the COVID-19 pandemic.

An important aspect of this pandemic is the dynamic and gradual nature of its infiltration into our lives. It took more than two months for pneumonia of unknown cause in Wuhan, which was first reported in detail by WHO on January 5, 2020 (World Health Organization, 2020), to be declared as a pandemic on March 11, 2020. Meanwhile, people started altering their healthcare behavior—from those who perceived greater risks from COVID-19 to those perceiving lesser risks. Our risk calculus is also driven in part by emotion and not just by the death toll from COVID-19. A celebrity's death sometimes has a significant impact on human behavior (Toriumi et al., 2020). Each key event has gradually changed people's perceptions and behaviors toward COVID-19. Japan, which faces China across the East China Sea and accepted 924,800 visitors from China in January 2020 (partly owing to the Chinese New Year on January 25) (Japan National Tourism Organization 2020), has been involved in this pandemic since the beginning. Therefore, Japan is an ideal place to study how diabetic patients have reacted and adapted themselves to the spread of COVID-19 since the beginning of the pandemic.

To provide further background during the pandemic, Japan's first patient of COVID-19 was reported on January 16, and the first large-scale infection with SARS-CoV-2, or cluster, was confirmed in the Diamond Princess (a cruise ship) on February 1. On February 27, all the Japanese public schools were closed. In response to the growing COVID-19 infection, a state of emergency was declared to Tokyo and several prefectures on April 7, was extended to the remaining prefectures in the following week and was lifted on May 25. Note, however, that the state of emergency only asks for voluntary restraint on nonessential outings and does not have coercive power to limit people's behavior, unlike lockdown. Japan marked the first-wave record of the daily COVID-19 deaths of 31 on May 2, and the second wave peaked at the end of July. Though the second wave was larger than the first wave, the government did not release the state of emergency (Nippon dot com n.d.). The number of deaths due to COVID-19 had been maintained at a very low level compared to many other countries for the first several months of the pandemic. The death toll due to COVID-19 in Tokyo and its surrounding three prefectures (Saitama, Chiba, and Yokohama) as of September 30, 2020 (when our data coverage ends) was only 719, or the death rate of 0.0019% (Nippon Hōsō Kyōkai n.d.; Statistics Bureau, Ministry of Internal Affairs and Communications 2021, Calculated by the authors.) In summary, it is unlikely that a government policy or an overwhelmed healthcare system coercively limits access to healthcare, although patients might have voluntarily refrained from doctor visits given their surroundings.

To investigate when the decline in routine medical care started, we first tested the hypothesis that H1: *doctor visits among diabetic patients started declining since January 2020, shortly after COVID-19 was first reported* by observing the trend of doctor visits of all diabetic patients. Thereafter, in terms of routine visits as a product of an individual's decision-making process, people with different backgrounds would react differently to COVID-19. Therefore, the second hypothesis posits that H2: *patients who are at a higher risk of severe COVID-19 were more likely to reduce the frequency of doctor visits*. Our data contain demographic, socioeconomic, and vital variables, each of which is more or less associated with the risk factors of COVID-19. We utilized these variables as touchstones to uncover the potential mechanisms underlying the differential behaviors.

## 2 Data and methods

### 2.1 Data

For our analysis, we rely on the data offered by a health insurance society in Tokyo, where primary policyholders work in the ground transportation industry. Japan's universal healthcare is provided by several systems, and one of these systems allows a group of companies (usually in the same industry) to become a joint insurer.

Specifically, we obtained insurance claims data from October 2016 to September 2020. Considering that insurance claims are reported monthly in Japan, we created a monthly panel dataset. For the unit of observation, we selected individuals diagnosed with diabetes by using diagnosis codes from 84,907 members whose enrollment status was confirmed as of March 31, 2020. There were 1110 and 107 individuals diagnosed with diabetes before and after April 1, 2018, respectively. Using the information of these 1217 patients, we created a monthly panel dataset ranging from April 2018 to September 2020. The 107 individuals diagnosed with diabetes after April 2018 were included in the sample after their first diagnosis, which resulted in 35,043 months–unit observations.

Among these patients, 201 patients who visited a doctor every month during the period of analysis and 37 patients with missing income values were excluded from the regressions with unit fixed effects, thus resulting in 979 patients and 28,401 month–unit observations in Table 1. To adjust the difference in the number of days across months in the statistical analysis, we once transform monthly data to daily data and hypothetically set all visits that were made in the middle of the month.

**Table 1:** Descriptive statistics.

	Mean	SD	Min	Max
Doctor Visits within a Month	0.677	0.468	0.000	1.000
MoCount	−6.069	8.771	−21.017	8.450
After·MoCount	1.354	2.496	0.000	8.450
Women	0.209	0.407	0.000	1.000
Dependent	0.144	0.351	0.000	1.000
Age as of March 31, 2019	57.5	8.46	24.0	73.0
Monthly Salary as of September 30, 2020	375,497	189,390	58,000	1,390,000
Dipeptidyl-peptidase IV inhibitor	0.353	0.478	0.000	1.000
Alpha-glucosidase inhibitors	0.086	0.281	0.000	1.000
Sulfonylurea	0.178	0.382	0.000	1.000
Biguanide	0.280	0.449	0.000	1.000
Thiazolidine	0.064	0.244	0.000	1.000
Glinide	0.025	0.155	0.000	1.000
Glucagon-like peptide-1 receptor agonists	0.025	0.158	0.000	1.000
Insulin	0.065	0.247	0.000	1.000
Sodium-glucose cotransporter-2 inhibitors	0.196	0.397	0.000	1.000
Compounding Agents	0.237	0.425	0.000	1.000
N of month–unit observations	28,401			

Data covered the period from April 2018 to September 2020.

Finally, a couple of supplemental remarks on this dataset. First, insurance claims are submitted by healthcare providers in Japan. Second, we do not have the records of the treatments for COVID-19, which are 100% covered by the government. However, COVID infection does not affect the membership status of the health insurance. On the other hand, our dataset does not distinguish those who dropped out from routine diabetic care and those who died of COVID-19 during April 2020 and September 2020. However, Japan's death rate due to COVID-19 was very low (≈0.002%) during this period. Therefore, a simple calculation in Appendix C shows that the expected number of deaths in our sample is only about 0.14, which is negligible.

### 2.2 Method for graphical analysis

To visually inspect the dynamic change in doctor visits after the emergence of COVID-19, we utilized the local mean smoothing technique with a triangular kernel and used a bandwidth of four. In other words, the weights for neighboring months linearly decrease and become zero before or after four months. The selection of the kernel does not affect the shape of local mean smoothing curves. The bandwidth was set at four because it is rare in Japan that the length of a prescription exceeds three months. The outcome variable, namely ${\text{Visit}}_{it}$, is an indicator of whether a patient i visited a doctor in a given month–year t. We code ${\text{Visit}}_{it}=1$ when at least one drug for diabetes was prescribed to the patient. Thus, we counted all visits made for diabetes treatment, but this does not exclude the possibility that the patient consulted a doctor for another illness at the same time.

### 2.3 Estimation

Regression analyses were performed to confirm the results of the graphical analyses controlling for various confounding factors. We first model the overall pattern of doctor visits with the full sample to test the first hypothesis that the doctor visits among diabetic patients started declining at the beginning of the COVID-19 pandemic by estimating the following logistic model:

(1) $$\begin{array}{l} P\left({\text{Visit}}_{it}=1|x_{it}\right)=\frac{\exp\left(f\left(x_{it}\right)\right)}{1+\exp\left(f\left(x_{it}\right)\right)}\\ f\left(x_{it}\right)=\beta_{1}{\text{MoCount}}_{t}+\beta_{2}{\text{After}}_{t}\cdot {\text{MoCount}}_{t}+\delta D_{it}+\alpha_{i}+\Phi_{t}, \end{array}$$

where the upper equation shows the relationship between the outcome and covariates, and the lower equation shows the content of the covariates. The outcome variable ${\text{Visit}}_{it}$ is defined in the previous subsection. ${\text{MoCount}}_{t}$ refers to the number of days as of the reference date, January 6, 2020, when the Ministry of Health, Labour and Welfare (henceforth, MHLW) of Japan first reported pneumonia of unknown cause (Ministry of Health, Labour and Welfare, 2020). Note that this value takes negative values before the reference date and is divided by 30 to interpret the results with respect to a month. We expect the doctor visits to decrease in this variable throughout the observed period because of the dropouts from regular outpatient care, most of which occurs within a few visits after the first diagnosis (Masuda et al., 2006). We also expect that the drop size decreases because more and more individuals in the sample become either regular outpatients or constant dropouts. Also, the coefficient of ${\text{MoCount}}_{t}$, or $\beta_{1}$, represents the average slope calculated during the pre-COVID period (Apr. 2018–Dec. 2019) after control.

By contrast, ${\text{After}}_{t}\cdot {\text{MoCount}}_{t}$ is our primary explanatory variable and the interaction term of between ${\text{After}}_{t}$ and ${\text{MoCount}}_{t}$, where ${\text{After}}_{t}$ is the dummy variable of whether the doctor visits were made after the first report of COVID-19 on January 6, 2020. Given that the first five days in 2020 include New Year's holidays and weekends, we coded ${\text{After}}_{t}=1$ for all doctor visits made in January 2020. Therefore, the coefficient of this variable indicates how much additional decrease in the rate of doctor visits since the emergence of COVID-19. The first hypothesis is supported if the coefficient is negative and statistically significant (i.e. $\beta_{2}<0$ or ${\text{OR}}_{\beta_{2}}<1$).

$D_{it}$ is a set of 10 indicators of whether a patient *i* was prescribed a given diabetes drug in the last visit. These 10 drugs include alpha-glucosidase inhibitors, biguanide, dipeptidyl-peptidase IV inhibitors, glinide, glucagon-like peptide-1 receptor agonists, insulin, sodium-glucose cotransporter-2 inhibitors, sulfonylurea, thiazolidine, and compounding agents. The values of these indicators were updated during the next visit. Therefore, $D_{it}$ represents the type of drug prescribed. We regard patients prescribed with insulin as being in a relatively advanced condition (American Diabetes Association, 2021c).

Finally, $\alpha_{i}$ and $\varphi_{t}$ are the fixed effects for patients and months, respectively. The month fixed effects ($\varphi_{t}$) enable us to make seasonal adjustments to doctor visits: human factors such as life and business cycles of the insured and environmental factors such as temperature, humidity, and pollution or allergen level in the air. The patient fixed effects ($\alpha_{i}$) control for the effects of all observed and unobserved individual characteristics that are constant during the period of analysis (i.e., October 2018–September 2020). Such characteristics include genes, socioeconomic environments determined prior to October 2018, and pre-existing illnesses. Given that logistic regression with the unit fixed effects yields biases with the ordinary maximum likelihood estimation, the models are estimated using a conditional likelihood approach.

To test the second hypothesis that patients at higher risk of severe COVID-19 were more likely to reduce the frequency of doctor visits, we analyzed the differential effects according to the following criteria: (1) men or women, (2) younger or older (defined as below or above the median), (3) higher or lower income (defined as below or above the median), and (4) never prescribed insulin or ever prescribed insulin. We regard those who fall in the second category (i.e., women, older patients, lower-income patients, and those prescribed insulin) as the target subsample and those who fall in the first category as the control subsample. As a proxy of patients' income, we use standard monthly remuneration, which is calculated from policyholders’ earnings and is used to determine the premium for the beneficiaries. Age and income were measured as of March 31, 2019, and September 30, 2020. To prioritize compatibility with visual evidence, age and income were dichotomized at their median values. Thus, we included the interaction term between each of the aforementioned four dummy variables and two other variables, ${\text{MoCount}}_{t}$ and ${\text{After}}_{t}\cdot {\text{MoCount}}_{t}$, in Eq. (1) in the model:

(2) $$\begin{array}{l} f\left(x_{it}\right)=\beta_{1}{\text{Ctrl}}_{i}+\beta_{2}{\text{Ctrl}}_{i}\cdot {\text{MoCount}}_{t}+\beta_{3}{\text{Ctrl}}_{i}\cdot {\text{After}}_{t}\cdot {\text{MoCount}}_{t}\\ \;\;\;\;\;\;\;\;\;\;+\beta_{4}{\text{Trgt}}_{i}+\beta_{5}{\text{Trgt}}_{i}\cdot {\text{MoCount}}_{t}+\beta_{6}{\text{Trgt}}_{i}\cdot {\text{After}}_{t}\cdot {\text{MoCount}}_{t}\\ \;\;\;\;\;\;\;\;\;\;+\delta D_{it}+\alpha_{i}+\Phi_{t}, \end{array}$$

where ${\text{Ctrl}}_{i}$ and ${\text{Trgt}}_{i}$ are the dummy indicators of whether a patient belongs to the control subsample and the target subsample, respectively. The subscript t is added to these indicators when the sample is divided on the basis of insulin prescription because some patients started taking insulin during the observed period. The coefficients of the triple interaction terms, namely, $\beta_{3}$ and $\beta_{6}$, represent the additional change in the rate of doctor visits after the emergence of COVID-19 for each of the control and target groups, which are the subsample equivalent of $\beta_{2}$ in Eq. (1).

## 3 Results

Table 1 presents the descriptive statistics of all variables used in our regression analysis. The first row shows that the mean of doctor visits within a month is 0.677, thus suggesting that two-thirds of patients saw a doctor at least once per month. The minimum and maximum of MoCount in the second row roughly correspond to the middle of April 2018 and September 2020, respectively. As discussed earlier, the drug variables starting from the eighth row refer to the type of drug prescribed in the last visit, and they show a low proportion of insulin and high proportions of sulfonylurea, biguanide, sodium-glucose cotransporter-2 inhibitor, and compounding agents.

### 3.1 Full-sample results

**Fig. 1:**
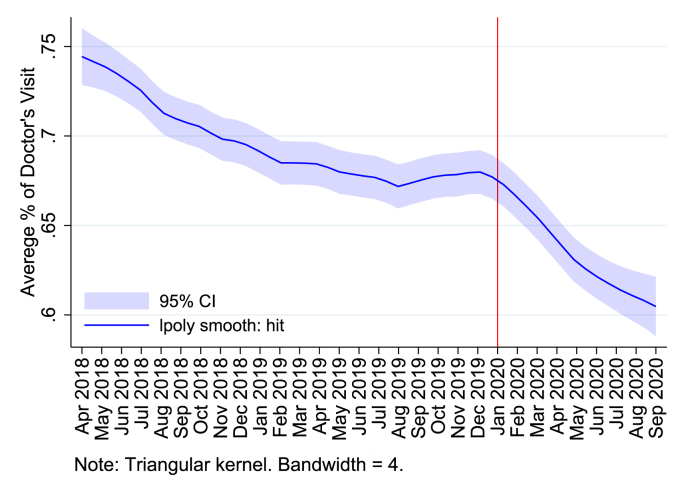
The change in the proportion of doctor visits using the full sample of people diagnosed with diabetes. The curve is drawn with the local mean smoothing of doctor visits from April 2018 to September 2020. The red vertical line indicates January 1, 2020. (For interpretation of the references to colour in this figure legend, the reader is referred to the Web version of this article.)

Fig. 1 shows how the emergence of COVID-19 caused behavioral changes among diabetic patients in Japan; the vertical axis represents the average proportion of patients with diabetes who visited a doctor's office at least once in a given month. The curve of local mean smoothing started declining after the emergence of COVID-19, thus indicating that patients visited doctors less frequently after the emergence of the disease.

Note that different mechanisms caused the decline in doctor visits during the first several months and the second one in 2020. The former was caused by the dropouts of newly diagnosed diabetic patients and the hot summer in 2018. Indeed, a study with diabetic patients in Japan showed that only one in ten individuals diagnosed with diabetes routinely visited a doctor and most patients dropped out within three or fewer visits (Masuda et al., 2006). We discuss this point in more detail in Appendix B.

We performed regression analysis to confirm this visual pattern while statistically controlling for various potential confounders. Table 2 reports the results of the logistic analysis with patient fixed effects with different sets of control variables (see Table A1 for the same table in coefficients and standard errors). The upper rows show the odds ratios, and the lower rows show the 95% confidence intervals. The odds ratios in the first and third rows in all columns show that the odds ratios remain almost unchanged regardless of whether we control for monthly dummies and last-prescribed drugs.

**Table 2:** Estimated effects of COVID-19 on doctor visits from April 2018 to September 2020 using the full sample of people diagnosed with diabetes.

Outcome: doctor visits	(1)	(2)	(3)
exp(*β*_1_): MoCount	0.984*∗∗∗* [0.978,0.990]	0.983*∗∗∗* [0.977,0.990]	0.982*∗∗∗* [0.976,0.989]
exp(*β*_2_): After*·*MoCount	0.969*∗∗∗* [0.950,0.987]	0.976*∗* [0.956,0.997]	0.975*∗* [0.954,0.996]
*H*_0_: *β*_2_ = 0	*p* = *.*001	*p* = *.*024	*p* = *.*018
Patient fixed effects	✓	✓	✓
Monthly dummies		✓	✓
Last-prescribed drugs			✓
N of units	979	979	979
N of month–unit observations	28,401	28,401	28,401

Conditional logistic regressions with heteroscedasticity robust standard errors clustered by patient ID. Top rows: odds ratios; bottom rows: 95% confidence intervals.

*∗ p < .*05, *∗∗ p < .*01, *∗∗∗ p < .*001 with a two-tailed *t*-test.

The odds ratios for $\beta_{1}$ and $\beta_{2}$ are both less than one and statistically significant across all models, thus suggesting that patients became less likely to see a doctor as time passes. The onset of the COVID-19 pandemic further reinforced this tendency. Note that although Fig. 1 shows the flat curve several months before the emergence of COVID-19, our estimate for $\beta_{1}$ represents the average slope until December 2019 after controlling for other covariates such as month and unit fixed effects. Therefore, the predicted downward trend of $\beta_{2}$ is not an artifact of this flat slope. According to model (3) with the full set of control variables, the odds ratios for ${\text{MoCount}}_{t}$ and ${\text{After}}_{t}\cdot {\text{MoCount}}_{t}$ are 0.982 and 0.975, respectively, thus implying that the average doctor visits for patients one month after COVID-19 emergence is 4.3 (= {(1 − 0.982) + (1 − 0.975)} × 100) percentage points(p.p.) lower than the outcome value predicted from the model in the month immediately before the emergence, and 2.5 p.p. is accounted for by the COVID-19 pandemic.

### 3.2 Subsample results

**Fig. 2:**
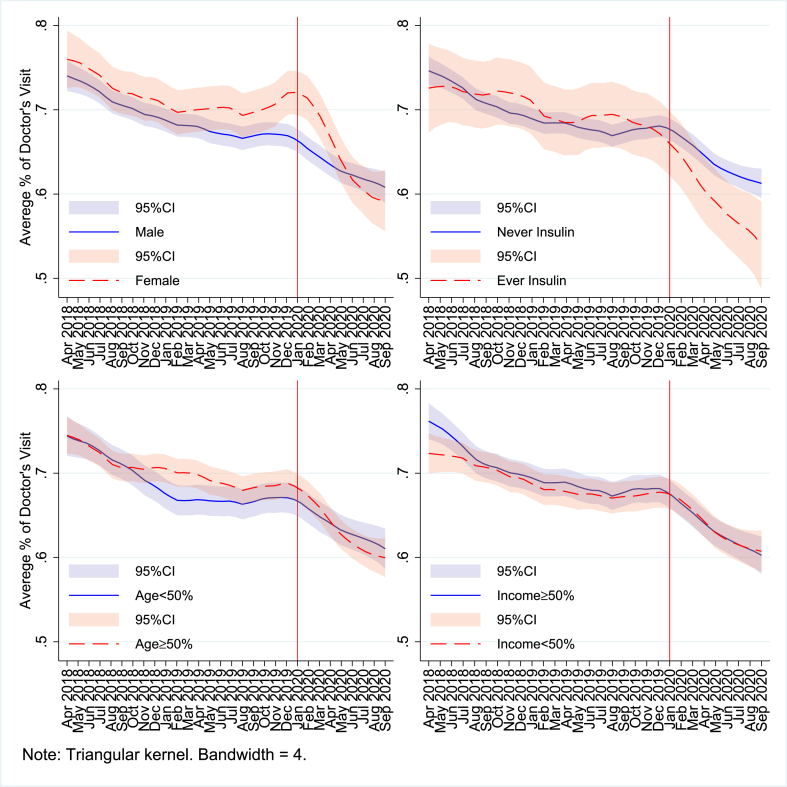
The change in the proportion of doctor visits using the subsamples. The subsamples were divided by sex (upper left), age (lower left), income (lower right), and diabetes progression (upper right). The curve is drawn with the local mean smoothing of doctor visits between April 2018 and September 2020. The red vertical line indicates January 1, 2020. (For interpretation of the references to colour in this figure legend, the reader is referred to the Web version of this article.)

We now focus on the differences of the trends between subsamples divided by sex, age, income, and progression of diabetes. We first analyzed the local mean smoothing plots to identify visual clues and then relied on regression analysis to test the hypotheses statistically. Fig. 2 shows the local mean smoothing plots of the differential effects of four key variables. The red dashed lines represent the local means of the target subsamples, whereas the blue solid lines refer to the counterparts of the control subsamples. The figure shows that the most significant reductions in doctor visits occurred in female patients (upper left) and those who were prescribed insulin (upper right). The older patients (lower left) show a moderate decline compared with the younger sub-population, whereas the division according to income shows no differential effects on doctor visits.

**Fig. A.1:**
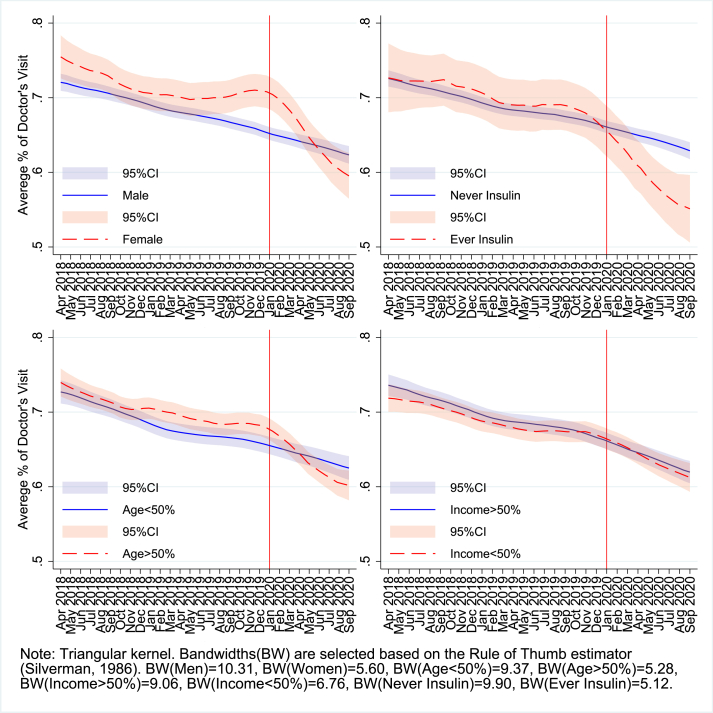
The change in the proportion of doctor visits using the subsamples with the Rule of Thumb bandwidth (Silverman, 1986). The subsamples are divided by sex(upper left), age(lower left), income(lower right), and severity of diabetes(upper right). The curve is drawn with local mean smoothing of doctor visits between April 2018 to September 2020. The red vertical line indicates January 1, 2020. See the footnote of the graph for the bandwidths used to draw each curve.

Table 3 examines these visual relationships statistically (see Table A2 for the same table in coefficients and standard errors). The differential effects of the emergence of COVID-19 based on sex, age, income, and diabetes progression are reported in the first to fourth columns. Overall, the results of the statistical tests correspond to the visual evidence. Specifically, ${\text{H}}_{0}:\;\beta_{3}=0$ was not rejected in all models at 5% level, thus suggesting that the additional decrease in medical care due to the emergence of COVID-19 was not confirmed in a statistical sense for the control subsamples. This point becomes more apparent when the local mean smoothing is drawn with the bandwidth selected according to the Rule of Thumb estimator (Silverman, 1986) to avoid over-fitting. Figure A1 shows that the curves of all control subsamples are drawn as a straight line.

On the other hand, ${\text{H}}_{0}:\;\beta_{6}=0$ was rejected in three models with $\beta_{6}<0$ or ${\text{OR}}_{\beta_{6}}<1$, thus indicating that those who belong to the target subsamples further reduced their doctor visits after the emergence of COVID-19. More specifically, the average visit one month later is 6.9 p.p. lower for female patients, 4.0 p.p. lower for older patients, and 7.7 p.p. lower for those who were ever prescribed insulin, excluding the impact of β5, or the temporal attrition of the doctor visits, than the average doctor visits before the emergence of COVID-19.

Finally, we tested whether the control and target subsamples reduced routine medical care at different paces after the emergence of COVID-19 by testing ${\text{H}}_{0}:\;\beta_{2}+\beta_{3}=\beta_{5}+\beta_{6}$. The sixth row from the bottom of Table 3 shows that the smallest *p*-value (p = .028) is in the first column, which examines the differential effects of sex. This is followed by insulin prescription (p = .040) and age (p = .094), although the difference in age was not statistically significant. Income does not have a differential effect (p = .939).

**Table 3:** Estimated effects of COVID-19 on doctor visits between April 2018 to September 2020 using the subsamples divided by sex, age, income, and diabetes progression.

Outcome: doctor visits	(1)	(2)	(3)	(4)
Control Subsample:	Men	Age < 50%	Income ≥ 50%	Never Insulin
Target Subsample:	Women	Age ≥ 50%	Age ≥ 50%	Ever Insulin

exp(*β*_1_): Ctrl	Subsumed	Subsumed	Subsumed	0.898
				[0.641,1.256]
exp(*β*_2_): Ctrl*·*MoCount	0.980*∗∗∗*	0.980*∗∗∗*	0.979*∗∗∗*	0.981*∗∗∗*
	[0.972,0.987]	[0.970,0.990]	[0.970,0.988]	[0.974,0.988]
exp(*β*_3_): Ctrl*·*After*·*MoCount	0.987	0.991	0.978	0.981
	[0.964,1.010]	[0.962,1.021]	[0.951,1.005]	[0.959,1.003]
exp(*β*_4_): Trgt	Subsumed	Subsumed	Subsumed	Subsumed
exp(*β*_5_): Trgt*·*MoCount	0.992	0.984*∗∗∗*	0.986*∗∗*	0.993
	[0.979,1.005]	[0.976,0.992]	[0.977,0.995]	[0.978,1.008]
exp(*β*_6_): Trgt*·*After*·*MoCount	0.931*∗∗*	0.960*∗∗*	0.972	0.923*∗∗*
	[0.892,0.972]	[0.934,0.987]	[0.943,1.001]	[0.876,0.972]

*H*_0_: *β*_3_ = 0	*p* = *.*266	*p* = *.*549	*p* = *.*112	*p* = *.*091
*H*_0_: *β*_6_ = 0	*p* = *.*001	*p* = *.*004	*p* = *.*057	*p* = *.*002
*H*_0_: *β*_2_ + *β*_3_ = *β*_5_ + *β*_6_	*p* = *.*028	*p* = *.*094	*p* = *.*939	*p* = *.*040
Patient fixed effects	✓	✓	✓	✓
Monthly dummies	✓	✓	✓	✓
Last-prescribed drugs	✓	✓	✓	✓
N of units	979	979	979	979
N of month–unit observations	28,401	28,401	28,401	28,401

Conditional logistic regressions with heteroscedasticity robust standard errors clustered by patient ID. Top rows: odds ratios; bottom rows: 95% confidence intervals.

*∗ p < .*05, *∗∗ p < .*01, *∗∗∗ p < .*001 with a two-tailed *t*-test.

## 4 Discussion

### 4.1 Substantive impact of the emergence of COVID-19

This study investigated the relationship between the emergence of COVID-19 and the frequency of doctor visits among diabetic patients with the following two hypotheses: (1) diabetic patients started reducing routine medical care after the emergence of COVID-19, and (2) this tendency was more prominent in the subgroups that were susceptible to COVID-19.

We first estimated the relationships between the emergence of COVID-19 and doctor visits by using the entire sample to test the first hypothesis. The visual and statistical evidence both indicated that diabetic patients started decreasing their doctor visits after January 2020. The magnitude of the impact is not negligible. The odds ratio for the additional reduction in doctor visits (${\text{After}}_{t}\cdot {\text{MoCount}}_{t}$) was 0.975, thus implying that the average rate of doctor visits decreases by 2.5 p.p. every month because of the spread of COVID-19.

### 4.2 Findings as expected

We then examined the differential effects of the emergence of COVID-19 to test the second hypothesis. Our findings contain expected and somewhat surprising results. Previous studies have shown that the elderly and diabetic patients are at a higher risk of severe COVID-19 (Zhou et al., 2020). Not surprisingly, the graphical and statistical pieces of evidence consistently showed that older patients and patients prescribed with insulin reduced their doctor visits at a higher pace than the younger patients and patients who were never prescribed insulin, although the impact on older patients is somewhat weak.

**Fig. 3:**
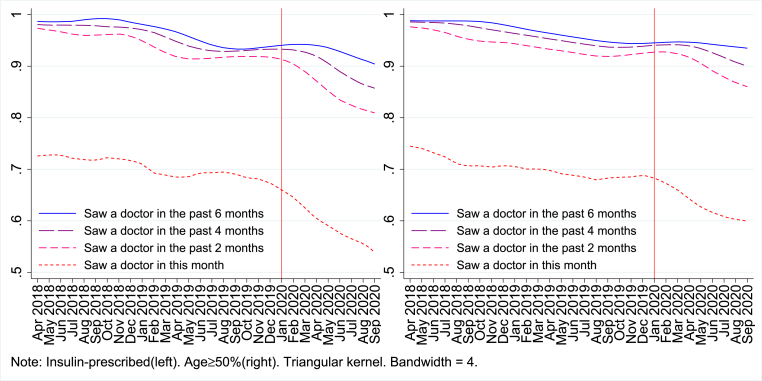
The change in the proportion of doctor visits for patients prescribed with insulin (left) and above median in age (right). The outcome variables take the value of “1” if a patient saw a doctor in a given month (red short-dashed line), within the past two months (pink dashed line), within the past four months (purple long-dashed line), and within the past six months (blue solid line). (For interpretation of the references to colour in this figure legend, the reader is referred to the Web version of this article.)

The reduction in doctor visits requires close attention if it implies the medically undesirable discontinuation of routine care, but it is a practical compromise if the interval of regular visits was widened following the doctor's advice. Fig. 3 was used to distinguish between these two possibilities. The left and right panels show the subsample smoothing plots for the insulin-prescribed patients and older patients respectively. The red short-dashed lines represent the reproduction in Fig. 2. The pink dashed lines, purple long-dashed lines, and blue solid lines are created by changing the outcome variable to whether a patient saw a doctor at least once in the past two, four, or six months, respectively.

Both panels show that the fraction of those who saw a doctor at least once in two or more months was quite high. However, we observed a similar reduction in the outcome variable after January 2020 in both panels when the outcome variables include whether a patient visited a doctor at least once in the past two or four months (i.e., the two dashed lines in the middle), thus implying that some patients were not able to visit a doctor once in four months. The sharp bends after the emergence of COVID-19 became visually negligible when the outcome variable was whether a patient visited a doctor at least once in the past six months. This indicates that patients with a high-risk profile for COVID-19 seemed to adapt to this crisis by setting an unconventionally long interval for doctor visits. For example, patients who require insulin probably consulted a doctor to obtain multi-month prescriptions because their health condition would be hard to sustain without insulin. These results imply that among the high-risk group, it is unlikely that the reduction in doctor visits caused an increase in mortality rates, which is also consistent with the finding that the dropout rate for diabetic patients who require insulin injection was much lower compared to diabetic patients with milder symptoms (Masuda et al., 2006).

These results imply that some diabetic patients probably took precautionary actions from an early stage of COVID-19 and relied on available information at that time rather than just waiting for the clinical risk evaluations of COVID-19 to become available around April 2020 (Fang et al., 2020; Guo et al., 2020). Indeed, information on the types of people who develop severe COVID-19 started circulating when Japan's first case was confirmed on January 16 (Ministry of Health, Labour and Welfare, 2020). The news was covered by a variety of media groups with background knowledge of past epidemic diseases. For example, a news article covering the first Japanese case already reported that “the infections of those who have chronic diseases and the elderly may become severe (Yomiuri Shimbun, 2020).” The first government expert meeting on February 16 attracted considerable media coverage, where advanced age and chronic illnesses, including diabetes, were clearly mentioned as the risk factors for COVID-19 (Asahi Shimbun, Yomiuri Shimbun).

It also comes as no surprise that virtually no differential effects were found between higher- and lower-income patients because income is not related to the exacerbation of COVID-19. At the same time, this finding highlights the importance of available healthcare options during a state of emergency to sustain routine care. If the universal healthcare system was not introduced in Japan, we might have observed differential effects between the rich and the poor because the availability and quality of healthcare are known to affect access to prompt doctor visits (Czeisler et al., 2020).

### 4.3 An unexpected finding and exploration

However, the analysis of the differential effects of sex revealed somewhat unexpected findings. From a medical perspective, the conditions of coronavirus-infected men are more likely to deteriorate than their counterparts (Gebhard et al., 2020; Scully et al., 2020). Therefore, male diabetic patients have more reasons to avoid routine medical care than women. However, our analysis revealed that the subsamples of women reduced doctor visits at a higher pace than their counterpart. Therefore, the differential effects between men and women stemmed from causes other than medical ones. What were they?

Although it is beyond the scope of this paper to test all potential causes, we propose one testable hypothesis: increased exposure to media coverage of COVID-19 discouraged female patients from visiting a doctor. During the pandemic, the show business industry was particularly hardhit. Lesser entertainment news became available, and news coverage of COVID-19 filled the gap. As Americans watched Dr. Fauci every day during the pandemic, several specialists appeared in Japanese TV shows every day to disseminate information about this disease. Moreover, the government's expert meeting asked citizens to refrain from going out for non-essential businesses as early as February 17, 2020 (Asahi Shimbun Feb. 17, 2020), thus further boosting the viewership of these programs.

**Fig. 4:**
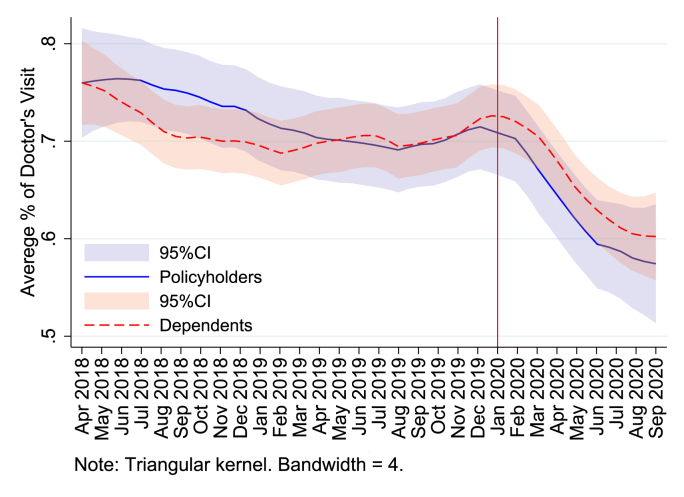
The differential effects of the emergence of COVID-19 on doctor visits with the subsamples of women. The subsamples are divided by membership status (policy-holder/dependent). The red vertical line indicates January 1, 2020. See the footnote of the graph for the bandwidths used to draw each curve. (For interpretation of the references to colour in this figure legend, the reader is referred to the Web version of this article.)

If the increased news intake is associated with less frequent doctor visits, female patients with a dependent status would have reduced their doctor visits more because they had more time to watch these news shows than female workers. Therefore, we analyzed the differential effects between policyholders and dependents within the subsample of women. Fig. 4 shows that the local mean smoothing curves look similar between these two groups, thus indicating the difference in news intake does not explain the women's reduced visits after the emergence of COVID-19. When we used Eq. (2) with the subsample of women and tested whether the size of the slope after the emergence of COVID-19 differs between female policyholders and female dependents, the null hypothesis ${\text{H}}_{0}:\;\beta_{2}+\beta_{3}=\beta_{5}+\beta_{6}$ cannot be rejected with *p* = .939.

The result defies our tentative proposition but agrees with a recent study that found no significant direct association between social media consumption and preventive behavior (Liu, 2021). Research on mass media also suggests that increased exposure to a particular media coverage cannot cause conforming behavior in its audience but may have limited yet diverse effects (Perse & Lambe,^2016^). It can be inferred that the reduction in doctor visits among female patients is related to their sex. A study reported that “higher rates of medication nonadherence due to costs” are seen among female diabetic patients in the United States (Bhuyan et al., 2018). However, an internet survey of US adults (Czeisler et al., 2020) and our study show that income inequality within female patients is unlikely to be a driver of reduced routine care. On the other hand, several studies show that women are more risk averse than men during this pandemic (Haischer et al., 2020) and in general (Jianakoplos & Bernasek,^1998^; Rosen et al., 2003). Given that pre-existing conditions often exacerbate COVID-19, it is not surprising that female diabetic patients responded to this pandemic in a highly risk-averse manner.

This study has several limitations. First, the exact dates of doctor visits and prescription lengths are missing to protect the privacy of the insured. This limitation may result in less accurate measurements. Second, readers may need to carefully evaluate the external validity of our findings. Our data come from a single joint insurer, where all members were under 75 years old and either they or their dependents worked in the ground shipping industry. Although we believe that this limitation might make some of the differentiating effects more conservative and would not change our substantive findings, the generalizability of the results might be compromised. Similarly, in the time horizon, it covers only the first several months after the emergence of COVID-19. Thus, we cannot examine the trajectories of their reduced doctor visits in the middle and long terms. Lastly, we cannot necessarily judge in this study whether fewer doctor visits reduce healthcare quality. The reduction in doctor visits among the older and insulin-prescribed patients due to the increased risk of infection was inevitable in this pandemic but was surely alarming because they require careful monitoring. By contrast, as suggested in Fig. 3, some patients and doctors might have arranged a reduced format of routine care without critically compromising healthcare quality.

## 5 Conclusion

In conclusion, our study contributes to the existing literature in two ways. First, our study fills the gap between the actual spread of COVID-19 and the healthcare behaviors of people. We show a dynamic reduction in routine medical care among diabetic patients after the emergence of COVID-19. Second, our study explains the factors that play an essential role in healthcare avoidance by using health insurance claims, which are behavioral-based measurements and augment the findings via opinion surveys (Czeisler et al., 2020). We found that lifestyle and economic factors have no significant impact, patients with high-risk factors have large behavioral changes, and sex-related factors play a crucial role. These findings facilitate a deeper understanding of human behaviors in response to this public health crisis, thus contributing to the improvement of communications with the target population, the delivery of necessary healthcare resources, and the provision of appropriate responses to future pandemics.

## CRediT authorship contribution statement

**Masataka Harada:** Conceptualization, Methodology, Software, Writing – original draft, Writing – review & editing, Visualization. **Takumi Nishi:** Software, Data curation, Writing – review & editing. **Toshiki Maeda:** Methodology, Writing – review & editing, Project administration. **Kozo Tanno:** Investigation, Resources, Writing – review & editing. **Naoyuki Nishiya:** Investigation, Resources, Writing – review & editing, and. **Hisatomi Arima:** Writing – review & editing, Supervision, Project administration.

## Declaration of competing interest

None.

## Figures and Tables

**Table A.1:** Replication of Table 2 with regression coefficients and standard errors.

Outcome: doctor visits	(1)	(2)	(3)
Estimation Methods	Cond.FE	Cond.FE	Cond.FE
*β*_1_: MoCount	−0.016*∗∗∗*	−0.017*∗∗∗*	−0.018*∗∗∗*
	(0.003)	(0.003)	(0.003)
*β*_2_: After*·*MoCount	−0.032*∗∗∗*	−0.024*∗*	−0.025*∗*
	(0.010)	(0.011)	(0.011)

*H*_0_: *β*_2_ = 0	*p* = *.*001	*p* = *.*024	*p* = *.*018
Patient fixed effects	✓	✓	✓
Monthly dummies		✓	✓
Last-prescribed drugs			✓
N of units	979	979	979
N of month–unit observations	28,401	28,401	28,401

Conditional logistic regressions with heteroscedasticity robust standard errors clustered by patient ID. Top rows: coefficients, bottom rows: standard errors.

*∗ p < .*05, *∗∗ p < .*01, *∗∗∗ p < .*001 with a two-tailed *t*-test.

**Table A.2:** Replication of Table 3 with regression coefficients and standard errors.

Outcome: doctor visits	(1)	(2)	(3)	(4)
Estimation Methods	Cond.FE	Cond.FE	Cond.FE	Cond.FE
Control Subsample:	Men	Age < 50%	Income ≥ 50%	Never Insulin
Target Subsample:	Women	Age ≥ 50%	Income < 50%	Ever Insulin
*β*_1_: Ctrl	Subsumed	Subsumed	Subsumed	−0.108
				(0.171)
*β*_2_: Ctrl*·*MoCount	−0.021*∗∗∗*	−0.020*∗∗∗*	−0.022*∗∗∗*	−0.019*∗∗∗*
	(0.004)	(0.005)	(0.005)	(0.004)
*β*_3_: Ctrl*·*After*·*MoCount	−0.013	−0.009	−0.022	−0.019
	(0.012)	(0.015)	(0.014)	(0.011)
*β*_4_: Trgt	Subsumed	Subsumed	Subsumed	Subsumed
*β*_5_: Trgt*·*MoCount	−0.008	−0.016*∗∗∗*	−0.014*∗∗*	−0.007
	(0.006)	(0.004)	(0.005)	(0.008)
*β*_6_: Trgt*·*After*·*MoCount	−0.071*∗∗*	−0.040*∗∗*	−0.029	−0.081*∗∗*
	(0.022)	(0.014)	(0.015)	(0.027)

*H*_0_: *β*_3_ = 0	*p* = *.*266	*p* = *.*549	*p* = *.*112	*p* = *.*091
*H*_0_: *β*_6_ = 0	*p* = *.*001	*p* = *.*004	*p* = *.*057	*p* = *.*002
*H*_0_: *β*_2_ + *β*_3_ = *β*_5_ + *β*_6_	*p* = *.*028	*p* = *.*094	*p* = *.*939	*p* = *.*040
Patient fixed effects	✓	✓	✓	✓
Monthly dummies	✓	✓	✓	✓
Last-prescribed drugs	✓	✓	✓	✓
N of units	979	979	979	979
N of month–unit observations	28,401	28,401	28,401	28,401

Conditional logistic regressions with heteroscedasticity robust standard errors clustered by patient ID. Top rows: coefficients, bottom rows: standard errors.

*∗ p < .*05, *∗∗ p < .*01, *∗∗∗ p < .*001 with a two-tailed *t*-test.

**Fig. B.1:**
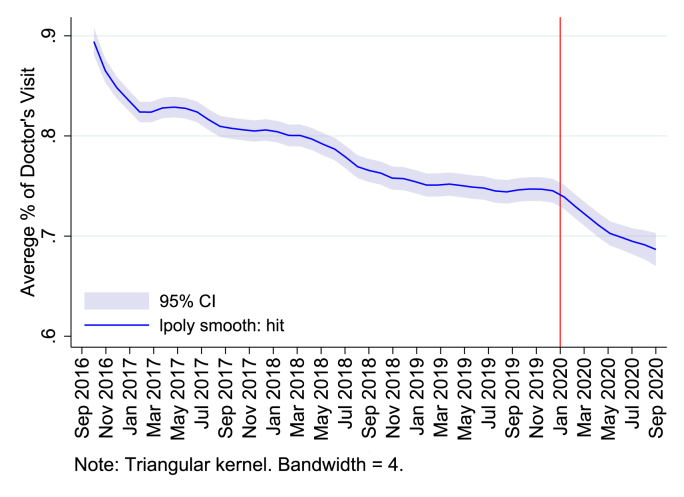
The change in the proportion of doctor visits using the individuals who joined the sample in October 2016 with the extended period of observation. The curve is drawn with the local mean smoothing of doctor visits from October 2016 to September 2020. The red vertical line indicates January 1, 2020.

**Fig. B.2:**
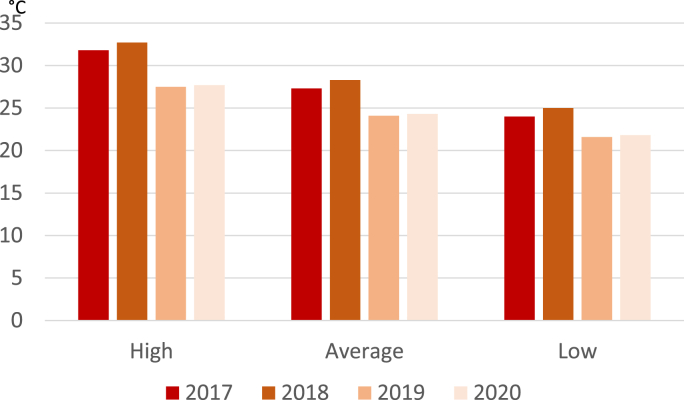
The bar charts of July's average temperatures in Tokyo from 2017 to 2020.

## Appendix B Explanation for the negative slope in 2018

In this appendix, we explain the reason why we observe the negative slope in Fig. 1. In short, this drop is explained by the combination of the dropouts from regular diabetic care and very high summer temperatures in 2018.

To illustrate these points, we drew Figure B1 below. This figure is different from Fig. 1 in that the sample is limited to the individuals who joined the sample in October 2016, and the starting date of the observation is now October 2016. One caveat is that the steep decline in the first several months (Oct.2016 to Jan. 2017) is caused by the coding of the outcome variable and should be ignored. Whenever individuals joined the sample, they were diagnosed with diabetes in doctor visits. Therefore, the outcome value of the first observation is always coded as one.

To look at the figure, although this sample primarily consists of regular outpatients or dropouts, we still observe a moderate decline over months. However, the slope in the pre-pandemic period is less steep compared with the post-pandemic counterpart, as well as the pre-pandemic slope in Fig. 1. This indicates that some amount of the decline in the pre-pandemic period can be explained by the dropout patients (Masuda et al., 2006). As we reported in Subsection 2.1, roughly 10% of the individuals joined the sample sometime after April 2018, and most of them became dropouts within several months. Therefore, as time passed, the proportion of dropouts (and a small number of regular outpatients) increased, so the curve also approached flat.

Second, a closer look at the slope suggests that the sharp declines occurred in July 2017 and 2018, not in July 2019 and 2020. We argue that this variation was likely to be caused by the difference in weather conditions in July. As we see in Figure B2, July temperatures in 2017 and 2018 were exceptionally high compared to 2019 and 2020. In Tokyo, where humidity is quite high in summer, individuals with diabetes may well skip their routine healthcare, especially if the condition is still not life-threatening. Indeed, the upper right panel of Fig. 2 shows no drop in July 2018 for the subsample for the patients who required insulin. These seasonal variations are statistically controlled for in the regression analysis using monthly dummy variables.

## Appendix C Estimation of COVID-19 deaths during April 1, 2020 and September 30, 2020

In this appendix, we calculate the expected number of death due to COVID-19 during the period when our data cannot identify whether the patients died of COVID-19 or did not see a doctor, namely from April 1, 2020, to September 30, 2020. The following calculation shows the estimated number of death due to COVID-19 is only 0.14, which is sufficiently small to ignore this issue in our inference.

In the following calculation, we relied on the following data sources: the COVID-19 data archive of NHK (Japan's national broadcasting company) for the death toll, the census for population, and the report by Ministry of Health, Labour and Welfare that was based on Japan's 322,007 COVID-19 positive patients for the death rates of COVID-19 positive people with and without diabetes (Nippon Hōsō Kyōkai n.d.; Statistics Bureau, Ministry of Internal Affairs and Communications n.d.; Ministry of Health, Labour and Welfare 2021).

Several caveats must be noted. First, our estimate is based on the statistics during the period mentioned above, and the area consists of Tokyo and its surrounding three prefectures (Saitama, Chiba, and Kanagawa), where almost all members of the health insurance society live. Second, the number of Japan's viral tests has been very low, so we relied on the number of COVID-19 death rather than the number of positive cases, which is likely to be underestimated. Third, our data cannot identify the death toll for other causes, but we statistically control for its impact, assuming the death rate of other causes is constant and not considered in this section. Finally, we assume that the risks of getting COVID-19 are the same regardless of whether one has diabetes or not.

The expected number of death due to COVID-19 was thus calculated as follows:

$$\begin{array}{l} \text{E}\left(\text{Death}\right)=\left(\text{Number of the Sample}\right)\\ \times \left(\text{COVID}19\text{ Death Rate for Diabetic Patients}\right)\\ =\left(\text{Number of the Sample}\right)\\ \times \left(\text{COVID}19\text{ Death Rate for the Population}\right)\\ \times \left(\text{Multiplier of COVID}19\text{ Death Rate for Diabetic Patients}\right)\\ =\left(\text{Number of the Sample}\right)\\ \times \frac{\text{Dearth Toll due to COVID}19}{\text{Population}}\times \frac{\text{Dearth rate of COVID}19\text{ positive People with Diabetes }}{\text{Dearth rate of COVID}19\text{ positive People without Diabetes}}\\ =979\times \frac{719}{36,938,977}\times \frac{0.0476}{0.0065}\\ \approx 0.1395. \end{array}$$

What this result tells us is, no matter how high the mortality risk due to COVID-19 is, our sample size is only 979, and only 719 people were dead due to COVID-19 by the end of September 2020 in the area of study. Therefore, the resulting estimate for the death toll is also very small.
